# Monthly Summary

**Published:** 1894-10

**Authors:** 


### Monthly Summary.

---

THE VALUE OF SUGAR AND THE EFFECT OF SMOKING ON MUSCULAR WORK.—As the result of a series of experimental researches in the Physiological Institute, Turin, as to the value of sugar and the effect of smoking on muscular work, Vaughan Harley (*Journal of Physiology*, vol. xvi, Nos. 1 and 2, March, 1894) has come to the following conclusions:

1. The periods of indigestion as well as the kind of food taken have a marked influence on voluntary muscular energy.

2. Irrespective of the influence of good, there is a periodical diurinal rise and fall in the power of performing muscular work.

3. More work can be done after than before midday.

4. The minimum amount of muscular power is in the morning about 8 a. m., the maximum about 4 in the afternoon.

5. Regular muscular exercise not only increases the size and power of the muscles, but has the effect of markedly delaying the approach of fatigue.

6. The amount of work performed on a diet of sugar alone is almost equal to that obtained on a full diet, fatigue, however, setting in sooner.

7. In fasting large quantities of sugar (500 grs.) can increase the power of doing muscular work during thirty voluntary contractions from 26 to 33 per cent. while the total gain in a day's work may be 61 to 76 per cent., the time before fatigue sets in being also lengthened.

8. The effect of sugar is so great that, when added to a small meal, it can increase the muscular power during thirty contractions from 9 to 12 per cent., while the total increase in work may be from 6 to 39 per cent., the approach of fatigue being at the same time retarded.

9. When added to a large mixed meal sugar can increase the muscular power of thirty contractions 2 to 7 per cent., the increase in total work being 8 to 16 per cent., and a marked increase in the resistance of fatigue is shown.

10. Two hundred and fifty grammes of sugar taken in addition to a full diet increases the day's work; the work accomplished during thirty voluntary muscular contractions shows a gain of from 6 to 28 per cent., the tolal day's work giving an increase of power 9 to 36 per cent., and the time before fatigue sets in being lengthened.

11. Moderate smoking, although it may have a slight influence in diminishing the power of doing voluntary muscular work, neither stops the morning rise nor, when done early in the evening, hinders the evening fall.

12. Sugar taken early in the evening is capable of obliterating the diurinal fall of muscular power that occurs at this time, and increases the resistance to fatigue.

---

WHY ARE WE WEARY.—Work and want of work, bring weariness. Hot haste and hot rooms bring fatigue. There are moral causes and there are physical—the moral produces the physical. But why do these do so? Dr. Hodge, of Clark University, has been for some years making experiments with a view of elucidating the pathology, or explaining the physiology, of fatigue. He refers to the fact that all the energy of the body comes directly from chemical changes and reactions in the individual cells of which the body is composed. If we could know the processes which take place in a single nerve cell, it would be the key to the whole of nerve physiology, from the action of the nervous mechanism in the protoplasm of the amœba, and thence through the entire animal series, to the activity of the human brain. It has been well known that every kind of cell has its peculiar function—gland cells to secrete their own fluid, the cells of muscle to produce work, whereas nerve cells produce what we call consciousness—a thing outside of physical observation.

But the nerve cells have the same life-history of birth, maturity, and death, as other cells; hence they suffer from want of nourishment, fatigue, and in the struggle for existence.

Dr. Hodge has been trying to find out what changes occurred in the cells of healthy animals; by passing electric currents through them. Various animals were used for this purpose, and after they were killed the nerve cells were examined under the microscope.

Some of the animals were put under the influence of ether, and a mild current carried directly to the one side of the optic thalamus and other sections of the brain, and after a time the animal was killed and opposite sections made and examined. The result of a large number of experiments exhibited a uniform diminution and decrease of the size of the nucleus of the cells, which had beed subjected to the stimulation, and a change from smooth and round to jagged, irregular outlines. The recticular appearance was broken up, the cell protoplasm was shrunken in size, and the nuclei were decreased in size. The sections of brain not stimulated showed no changes of this nature. Great care was taken that the electrical current should be within physiologic limits. This inquiry followed. Do similar changes occur in the normal activity of an animal from fatigue? and they are restored by rest and sleep? To answer this numerous sparrows, pigeons and swallows were shot in the morning, as they were going out, and at night when coming home after a day of activity. Sections of similar parts of the brains of the morning and evening birds were compared with each other. In all cases the brain cells of the birds at evening were greatly decreased in size, and the nucleus jagged and of irregular outline. The cell protoplasm was also shrunken and reticulated in appearance. The nuclei were also greatly decreased. Honey bees were caught coming out of the hive in the morning and compared with the bees going in late at night. The same differences were noted in the brain cells, showing degrees of exhaustion and fatigue in one, and vigor and health in the others.

These studies, conducted with great accuracy and detail for the purpose of eliminating all errors, seem to point to the brain and nerve fatigue as a shrinking of the nucleus and cell protoplasm. Birds identical in appearance, shot at night and in the morning, brought out this fact, in the shrunken cells at night, and the full rounded cells in the morning. The conclusion is that in sleep the wasted cell recovers its functional changes from fatigue, and the failure of this restoration is the beginning of organic degeneration of all forms and degrees. The cell recovers the bundles of nerve fibres which enter into it, and are lost in the granular substance and nucleus. Here all the impressions of the external world come in a succession of impulses, and all the outgoing commands are determined.

Something like this decreased and shrinking of cell contents and nucleus are common in old age. It would appear reasonable to suppose that failure to restore the cell contents would be ageing and practical starvation of the body. If physiological fatigue is functional changes in nerve cells which can be restored by rest and sleep, scientific inquiry has taken a step forward of deep significance.

Dr. Hodge has been for some time making studies of the changes which take place in nerve cells under variations of food and water supply. Also what changes if any take place in nerve cells from birth to death from old age, rom rejuvenation to senescence. These researches will be watched with much interest.—*The Popular Medical Monthly.*

---

EXTRACTING FOR IRREGULARITY.—The habit of extracting bicuspids or first molars to give space to protruding or crowding front teeth is bad and useless practice. Back teeth will move forward, but it is seldom that front teeth will move backward. Even the space of an extracted central will seldom be filled by its fellow; though the lateral on the same side may leave the cuspid and come forward. The extracting of third molars to give more space for first or second molars is scarcely ever of any use, though the removal of a first molar is likely to cause the second and third to move forward, if the subject is young. But the tipping of the second molar as it moves forward is so often conspicuous and inconvenient that this practice is generally objectionable.—*Items of Interest.*

---

Dr. J. Austin Dunn of Chicago has reduced the price of his Syringe with one Iridio-Platinum needle to $1.50. The I-P Needle is something the dentists have always wanted but could not procure, and is a step in advance. The iridium makes the needle stiff, so that it will wear smooth and is more lasting and durable. Another advantage is that it can be held in the flame of the spirit lamp and cleaned by burning out the debris without danger of softening it. The price for extra needles has been fixed at 50c so that they are within the reach of all.

AN ORIGINAL CROWN.—Dr. J. H. Crossland, of Montgomery, Ala., speaking of an original crown, says:

"For this crown a band is set in a groove cut in the end of the root as near the circumference as possible. To this a flat cap is soldered and burnished to the end of the root, exactly to the margin. This is pierced for a platinum pin to which is affixed a porcelain plate tooth with gold backing. The porcelain is ground at the cervical end to fit the gold cap, and beveled from the pins to the cutting edge, over which the first backing is burnished. A second backing is added, which is punched full of holes and soldered to the first to give added body and strength. If preferred, a platina cap and backing can be used and porcelain backing baked to the crown, making a very artistic crown."

Dr. Crossland claims that this crown is less bulky and more shapely than the old ferrule crown; exposes no gold at gum margin; allows a much more favorable and accurate adjustment with regard to the position of other teeth; can be used upon strikingly convergent or divergent roots which are to serve as abutments for bridges; is especially adapted to conically shaped roots; causes less pain in adjustment and will not irritate the pericemental membrane. It is extremely cleanly, as the joint between gold and tooth can be made about perfect.—*Southern Dental Journal and Luminary.*

---

TO BAND A LOGAN CROWN.—Prepare tooth as for Richmond crown; grind Logan crown proximately to fit root; make gold band 22k., 29g., extra wide and larger at crown opening than at root. Place band on root and grind crown to fit opening of band tightly, and drive crown home with mallet; remove band and crown together, and with corundum stone grind away band in front, leaving it very long in back; grinding and burnishing it over the bulbous portion. Grinding toward the tooth will burnish the band to the tooth, and a perfect joint will be made. Requires no inside disk and no soldering. It is strong and durable. The tooth can be banded more quickly than a Logan crown can be adjusted, by grinding to fit the abutment of the root closely.—*Items of Interest.*

DEVITALIZING PULPS.—An article in a recent copy of *The Dental Office and Laboratory* says, "The uncertainty attending the use of arsenic for devitalizing pulps often makes its use unpleasant and unsatisfactory." The writers experience and my own are at variance. It is the rarest thing in my practice, that for arsenic paste (the ordinary kind furnished for us by the dealers), not to do all for me that I expect of it. It has never proven "uncertain," when I was satisfied with the application. The method of applying the paste has much to do with the result. There must be access and exposure. I always see the pulp either direct or by reflection. Then I thoroughly dry the cavity and apply directly on the pulp two or three small crystals of hydrochlorate of cocain, waiting for exudation from the pulp to dissolve the crystals. Then put the arsenic in direct contact with the pulp, a little pressure not giving pain because of its anethetized condition. Cover the paste lightly, filling the cavity with dry cotton and sandarac varnish, letting the paste remain ten hours.

If those practitioners who insist on two or four days, application would try the ten-hour plan on a direct exposure, they would have less peridental inflammation. The reason for desiring a good exposure is because, when there is inflammation of the pulp, there is swelling. which gives rise to the intense pain following the ordinary application of arsenic. Remove one of these walls and you mitigate the pain.—*Georgia Practitioner.*

---

TO OBTUND SENSITIVE DENTINE.—Put the patient on an alkaline treatment for several days previous to the operation. Put a teaspoonful of soda in a glass of water, and have the patient hold a little in the mouth frequently through the day —perhaps twenty or thirty times. The next day double the proportion of soda, and again the third day. The fourth day pack soda in the cavities for some little time before cutting he tooth, and you can then cut without pain. Soda is the natural tooth obtundent.—*Items of Interest.*

PULPITIS.—Remove loose decay. washing out the cavity with warm water, dry and apply equal parts of arsenic and tannin, finely powdered, and mixed with a syrup solution of cocain and carbolic acid. Retain this with a pellet of cotton over which flow thin cement, gently pressing and wiping off the latter with a wet pellet of cotton. Fill next day, but before removing the dead pulp carefully work the cocain and carbolic acid down to the end of root canals with a Donaldson bristle. It will prevent sensitiveness and hemorrhage.—*C. H. Thorn.*

---

BLOOD POISONING.—A peculiar case of blood-poisoning which caused quite a sensation among the dentists of Berlin. Dr. Bernheim, a dentist of Berlin, extracted a tooth for a lady and got his finger into her mouth, whereupon she closed her teeth with a convulsive firmness on his index finger. After a few hours symptoms of blood-poisoning appeared on the doctor's hand and spread so rapidly that even an immediate amputation did not check the spreading. He became delirious and, notwithstanding the efforts of two physicians, Dr. B. died the following day.—*Berlin Journal.*

---

MOUTH POULTICE.—Dr. Hugenschmidt recommends as the only practical poultice that can be used in alcolar abscess a fig boiled in a solution of boric acid and cut in halves, and the cut surfaces sprinkled with powdered boric acid. It will in most cases cause the abscess to discharge into the mouth. Should the abscess be far advanced and threaten to break through the cheek, then apply an ice-compress with the above.—*Dominion Dental Journal.*

